# Utility of the cellular energy allocation model for assessing food limitation stress in freshwater mussels

**DOI:** 10.1093/conphys/coaf086

**Published:** 2025-12-18

**Authors:** Kaelyn J Fogelman, Andrea K Darracq, Monte A McGregor, James A Stoeckel, Wendell R Haag

**Affiliations:** School of Renewable Natural Resources, Louisiana State University and Agricultural Center, 227 Renewable Natural Resources Building, Baton Rouge, LA 70808, USA; Department of Biological and Environmental Sciences, Troy University, 498 University Avenue, Troy, AL 36082, USA; Department of Biological Sciences, Murray State University, 2112 Biology Building, Murray, KY 42071, USA; Watershed Studies Institute, Murray State University, 2112 Biology Building, Murray, KY 42071, USA; Kentucky Department of Fish and Wildlife Resources, Center for Mollusk Conservation, 3761 Georgetown Road, Frankfort, KY 40601, USA; School of Fisheries, Aquaculture, and Aquatic Sciences, 203 Swingle Hall, Auburn University, Auburn, AL 36832, USA; Southern Research Station, Center for Bottomland Hardwoods Research, US Forest Service, 3761 Georgetown Road, Frankfort, KY 40601, USA

**Keywords:** Biomarkers, carbohydrate, cellular energy allocation, electron transport system, lipid, protein, unionid

## Abstract

Freshwater mussel populations are declining worldwide, but the causes and mechanisms of these declines are poorly understood. Biomarkers that reflect the health or fitness of individual mussels are needed for understanding causes of mussel declines, but existing approaches each have weaknesses. We conducted two laboratory experiments to examine the utility of the cellular energy allocation (CEA) model for assessing juvenile mussel responses to stress induced by food limitation. The CEA assesses the energetic status of an organism as CEA = *E*_a_/*E*_c_, where *E*_a_ is available energy reserves (total carbohydrates, protein, and lipids) and *E*_c_ is energy consumption, estimated using electron transport system (ETS) activity as a proxy for respiration rate and metabolic demands. Experiment 1 evaluated the effects of food abundance (fed and unfed) on CEA and its component biomarkers at a single temperature (mean = 26.8°C) over 23 days. Experiment 2 evaluated the response of ETS activity to food abundance (unfed, low food, high food) in relation to temperature (20, 25, 30°C) over 27 days. In Experiment 1, most constituent biomarkers were lower in unfed mussels, but CEA did not differ between treatments because *E*_a_ and *E*_c_ declined by similar magnitudes. In Experiment 2, ETS declined with decreasing food abundance, but only at 25 and 30°C, and ETS was affected by temperature only in the unfed treatment. The ETS enzyme assay can be an informative biomarker of stress, but it requires accounting for confounding factors such as food, temperature, and species identity, as well as the lag time in response of ETS relative to respiration rate. Despite its value as a robust, holistic stress biomarker in other organisms, CEA may have limited usefulness for bivalves because of their tendency to reduce feeding and energy consumption under stress, which results in a simultaneous decline in *E*_a_ and *E*_c_.

## Abbreviations

CEA: cellular energy allocation
*E*_a_
: Energy available (sum of total carbohydrates, protein, and lipids)
*E*_c_
: Energy consumption (estimated with electron transport system activity)
ETS: Electron transport system

## Introduction

Freshwater mussel populations (order Unionida) are declining worldwide, but the causes and mechanisms of these declines are poorly known (FMCS, 2016; Böhm *et al.,* 2021; Aldridge *et al.,* 2023). A critical research need in understanding mussel declines is developing biomarkers that reflect the overall health status or fitness of individual mussels. Such biomarkers are needed to allow evaluation of sublethal mussel responses to a wide array of potential stressors (Newton and Cope, 2006). Many biomarkers have been used to assess mussel health, including growth, condition indices, energy stores (e.g. carbohydrates, lipids, protein), metabolomics and transcriptomics, endocrine responses, histopathology, and valve activity, but each has weaknesses (Newton and Cope, 2006; Waller and Cope, 2019).

Growth is used commonly because it is easy to measure and may be a sensitive indicator of health in some situations (Haberle *et al.,* 2025). However, mussel growth is strongly influenced by intrinsic environmental factors such as temperature, nutrient availability, and alkalinity, which can confound interpretations of health, and growth is not a useful marker for slow-growing adults (Haag *et al.,* 2019). As a primary short-term energy storage molecule, glycogen can be a sensitive indicator of stress (Hummel *et al.,* 1989; Naimo *et al.,* 1998; Patterson *et al.,* 1999), but variation due to environmental conditions and among species limits its usefulness as a robust biomarker (Haag *et al.,* 1993; Payton *et al.,* 2016; Hornbach *et al.,* 2021). As a long-term energy storage molecule, lipids may not be a consistently responsive biomarker (Haag *et al.,* 1993; Abad *et al.,* 1995). Metabolomics offers the ability to detect changes in specific biochemical pathways (Wishart, 2019), and this approach has been used to evaluate stress responses in mussels (Roznere *et al.,* 2014, 2017; Fritts *et al.,* 2015; Putnam *et al.,* 2023; Waller *et al.,* 2023). However, the high cost, expertise required, and vast array of potential response profiles limit its use as a routinely employed biomarker (Alonso *et al.,* 2015; Pinu *et al.,* 2019; Waller and Cope, 2019). An ideal biomarker would combine the comprehensive approach of metabolomics with the cost-effectiveness and accessibility of traditional measures.

The cellular energy allocation (CEA) model is an approach for assessing the fitness of an organism based on its energetic status. The CEA model quantifies the energy budget as CEA = *E*_a_/*E*_c_, where *E*_a_ is available energy reserves (total carbohydrates, protein, and lipids) and *E*_c_ is energy consumption, estimated using electron transport system (ETS) enzyme activity as a proxy for respiration rate and metabolic demands (De Coen and Janssen, 1997). Stress is typically indicated by a decrease in the net energy budget (lower CEA), which can be caused by a reduction in food or energy availability (lower *E*_a_), an increase in energy consumption (increased *E*_c_), or a synergistic effect of simultaneously reduced *E*_a_ and increased *E*_c_. The CEA model has been used as a stress biomarker for numerous aquatic organisms including daphnids (De Coen and Janssen, 1997, 2003; Muyssen *et al.,* 2002), fishes (Abe *et al.,* 2018; Damian *et al.,* 2019), and bivalves (Smolders *et al.,* 2004; Erk *et al.,* 2011; Wang *et al.,* 2012; Shang *et al.,* 2021). By integrating multiple biochemical markers, CEA has the potential to provide a more holistic assessment of health or stress than single markers alone.

A possible limitation of CEA is that while it directly measures energy reserves, it uses ETS activity as a proxy for energy consumption. ETS is interpreted as an estimate of the maximum potential respiration rate given existing enzymatic capacity, and it does not represent the actual organismal respiration rate (Simčič *et al.,* 2014; Bielen *et al.,* 2016). ETS activity is typically highly correlated with metabolic rate (energy consumption), but changes in ETS activity may lag behind changes in respiration rate (Båmstedt, 1980; Madon *et al.,* 1998). In addition, ETS is sensitive to confounding factors such as temperature, food quality, and food quantity (Fanslow *et al.,* 2001; Bielen *et al.,* 2016). For example, the relationship between ETS activity and temperature may follow a different trajectory than that between respiration and temperature (Hussain *et al.,* 2023). Such confounding factors would affect the overall energy budget measured by CEA, potentially making it an unreliable biomarker across a range of conditions of temperature and food.

We examined the utility of CEA and its constituent biomarkers for assessing mussel responses to stress induced by food limitation. We conducted two laboratory experiments. First, we exposed juvenile mussels to two food treatments, fed and unfed, and assessed the ability of CEA and individual biomarkers to differentiate the two groups. Second, we conducted a factorial experiment to simultaneously examine the effects of food availability and temperature on ETS activity to evaluate how those factors interact to influence ETS. For Experiment 1, we hypothesized that CEA of unfed mussels will be lower than that of fed mussels. For Experiment 2, we hypothesized that ETS will increase with increasing temperature and food availability. We discuss how our results inform the utility of CEA for assessing mussel health and stress responses.

## Materials and Methods

### Experiment 1: testing the ability of CEA and constituent biomarkers to distinguish fed from unfed mussels

We cultured juvenile *Lampsilis cardium* larvae from broodstock collected from the Rockcastle River, Kentucky, USA. Larvae (glochidia) of most mussel species are parasites that require a fish host to metamorphose to the juvenile stage. We inoculated *L*. *cardium* glochidia on Largemouth Bass (*Micropterus nigricans*) in January 2018, held inoculated fishes in a recirculating aquarium system at 19–23°C, and collected metamorphosed juvenile mussels 3–4 weeks after inoculation. We reared juveniles for approximately 4 months in a greenhouse with natural lighting at 24–26°C in 5.8-l aerated trays within a recirculating aquaculture system with biological and mechanical filtration (see White *et al.,* 2022 for details). Flow rate through each tray was maintained at 100 ml·min^−1^, and each tray received 50 ml of 150- to 250-μm heat-sterilized sand distributed evenly across the bottom. The system was filled with water purified by reverse osmosis then mixed with well water to an alkalinity of 60–80 mg CaCO_3_·l^−1^ and pH of 8.1. We fed juveniles a mixture of commercial and cultured algae via a feeding cone and automated ball valve, which delivered food to the trays continuously. The feeding ration used to rear juvenile mussels was identical to the fed treatment during the experiment (see subsequent).

We conducted a feeding experiment in the same system used to rear juvenile mussels. We established two feeding treatments: fed and unfed. Fed mussels received a ration of 10.6 mg algal dry mass·l^−1^·day^−1^ consisting of two live cultured algae (*Chlorella sorokiniana*, 3.4 mg·l^−1^; *Phaeodactylum tricornutum*, 2.2 mg·l^−1^), two commercially available concentrates of dead marine algae (*Thalassiosira pseudonana*, TP 1800, 2.3 mg·l^−1^; *Nannochloropsis* spp., Nanno 3600, 1.8 mg·l^−1^), and 0.9 mg·l^−1^ of a commercially available mixture of six dead marine microalgae (Shellfish Diet 1800; all marine algae from Reed Mariculture Inc., Campbell, California, USA). Unfed mussels received no food.

We established three trays for the fed treatment and three trays for the unfed treatment, and each tray received 30 haphazardly selected juvenile mussels. We randomized placement of treatment trays within a 1 × 6 array on the greenhouse shelving. We initiated the experiment on 20 June 2018, at which time individual mussels averaged 0.067 g of blotted wet mass (including shell; mean shell length = 8.3 mm ± 0.9 SD). Mean water temperature throughout the experiment was 26.8°C ± 0.5 SD. We examined all trays for mortality every 5–7 days. To ensure that we were able to capture a biomarker response to the treatments in the event that we experienced a widespread mortality event, we removed a subset of five individuals from each tray on June 27 and again on July 5, weighed them, and froze them immediately at −80°C. Mortality was first observed on July 10 (unfed treatments only, see Results), at which time we weighed all surviving mussels, removed and froze 10 mussels from each tray, and returned the remaining mussels to the trays. We terminated the experiment on July 13 (day 23) and weighed and froze all remaining surviving mussels. We calculated instantaneous mortality (Ricker, 1975) between all successive pairs of examination dates to account for removal of individuals during the experiment. We expressed mussel growth in each experimental tray as instantaneous growth (day^−1^, ln[final mass in g/initial mass in g]; Ricker, 1975) based on mean individual mussel mass in each tray at the beginning and end of the experiment (excluding individuals removed during the experiment).

We thawed mussels on ice and dissected the tissue from the shell. For each individual, we minced the tissue finely with a sterilized razor blade and placed a 10-mg subsample of tissue into a 2-ml microcentrifuge tube with three to four 1-mm homogenization beads and 1 ml of 4°C ultrapure water. If individual mussels weighed <10 mg, we pooled individuals from the same experimental tray to achieve a sample mass of 10 mg. We homogenized each sample on a beadbeater (BioSpec Products® Mini-BeadBeater-16) for 20 s, placed the samples on ice for 1 min, and repeated these steps two additional times. We centrifuged the samples for 5 min at 3000 rpm and placed them on ice. We transferred 400, 100, and 100 μl of supernatant from each vial into 1.5-ml microcentrifuge tubes for carbohydrate, protein, and ETS assays, respectively, and we used the tissue remaining in the 2-ml tube for the lipid assay. We stored the five vials associated with each sample at −80°C until assays were run. We measured the total carbohydrates (mg·mg WW^−1^) following Bove *et al.* (2022), modified as follows. We used two stock solutions of 1 and 10 mM l(−)-glucose (Acros Organics 921-60-8) to create a standard curve consisting of a blank and nine standards that varied in concentration from 0.009 to 0.901 mg·ml^−1^ l(−)-glucose. We thawed samples on ice and added 300 μl of sample to 700 μl of 4°C ultrapure water in a 12 × 75 mm glass vial to create a 1.0:3.3 dilution. We vortexed samples for 5 s, added 25 μl of 5% phenol, vortexed samples again for 5 s, and immediately added 2.5 ml of concentrated sulfuric acid (95% certified ACS grade). We incubated the samples for 1 min at room temperature (~22.2°C) and transferred them to a room temperature water bath where we incubated them for 30 min. After incubation, we vortexed each sample for 5 s and then aliquoted 200 μl of each standard and sample into a 96-well plate in duplicate. We read the plates on a spectrophotometer (BioTek® 800 TS) at an absorbance of 490 nm.

We measured total protein (mg·mg WW^−1^) using a Micro BCA Protein assay kit (Thermofisher 23235). We created the standards and working reagent following the kit protocol and diluted samples to 1.0:32 by adding 10 μl of the sample to 310 μl of ultrapure water. We pipetted 150 μl of each standard and sample into a 96-well microplate in duplicate. We then added 150 μl of working reagent into each well and mixed the plate on a plate shaker for 30 s. We covered and incubated the plate at 37°C for 2 h, allowed the plate to cool to room temperature, and read the plate on a spectrophotometer at an absorbance of 562 nm.

We measured total lipids (mg·mg WW^−1^) following Weber *et al.* (2020), modified as follows. We added 400 μl of chloroform and 200 μl of methanol to each 2-ml tube containing 10 mg of the remaining sample tissue. We added 160 μl of 0.05 M NaCl to each vial, capped the vial and gently inverted it twice, then uncapped and recapped the vial. We centrifuged the vials at 3000 rpm for 5 min and removed and discarded the upper phase. We placed the lower phase into a 12 × 75 mm glass vial. We prepared a stock solution of 1.5 mg·ml^−1^ corn oil (Supelco 47112-U) to create a standard curve consisting of a blank and eight standards ranging from 0.007 to 0.900 mg·ml^−1^ corn oil. We evaporated the standards and samples at 90–100°C until <20 μl remained, then added 5 ml of a vanillin–phosphoric acid solution to each vial (120 mg vanillin, Thermo Scientific Chemicals 14 082, 20-ml ultrapure water, and 80 ml of 85% phosphoric acid). We incubated the samples at room temperature for 10 min, vortexed them for 5 s, and then aliquoted 200 μl of each standard and sample into a 96-well plate in triplicate. We read the plates on a spectrophotometer at an absorbance of 562 nm. We estimated the total carbohydrate, protein, and lipid content of each sample based on each assay’s respective standard curves, adjusted to the blanks, and multiplied them by the dilution factors described above.

We measured the ETS activity based on Packard (1971) and Simčič *et al.* (2014), modified as follows. We diluted samples to 1 mg·ml^−1^ of sample with ultrapure water. For each sample, we filled three wells of a 96-well plate with 150 μl of buffered substrate solution (0.1 M sodium phosphate buffer, 1.7 mM NADH, 0.25 mM NADPH, 0.2% v/v Triton-X-100, and reagent grade water). Then, we added 50 μl of each sample to two of these wells and used the third well as a blank. We started the reaction by adding 50 μl of 2.5 mM *p*-iodonitrotetrazolium to all wells followed by incubation in the dark for 30 min at 27°C. After 30 min, we added 50 μl of stop solution (1:1 formaldehyde and 85% phosphoric acid) to each well. We immediately added 50 μl of each sample to its respective blank well and measured the production of formazan on a spectrophotometer at an absorbance of 490 nm. We estimated ETS activity for each sample as ml O_2_·g^−1^·h^−1^ following Simčič *et al.* (2014).

We converted all measures of constituent biomarkers to energy availability (as mJ·mg^−1^) using energy equivalencies of 17 500, 39 500, and 24 000 mJ·mg^−1^ for carbohydrates, lipids, and proteins, respectively (Gnaiger, 1983). For ETS, we calculated the cellular oxygen consumption rate based on the stoichiometrical relationship between formazan production and oxygen consumption following Gnaiger (1983) and summarized as follows. For every 2 μmol of formazan produced during the ETS assay, we considered that 1 μmol of oxygen was consumed. We then converted ‘ml O_2_·g^−1^·h^−1^’ into energetic equivalents using the ideal gas law to convert to ‘mg O_2_ g^−1^·h^−1^’ and then multiplied each value by 14.06 mJ·mg O_2_^−1^, which is the specific oxyenthalpic equivalent for an average lipid, protein, and carbohydrate mixture (Gnaiger, 1983). From this, we calculated the ‘mJ·mg^−1^’ of energy available (*E*_a_) as the sum of carbohydrate, lipid, and protein energy, and the energetic cost rate (*E*_c_: mJ·mg^−1^·h^−1^) as the converted ETS activity. We calculated CEA as *E*_a_/*E*_c_ (Verslycke *et al.,* 2004)_,_ which is a unitless measure representing the proportion of available energy stores that would be consumed in an hour at a maximum metabolic rate. In addition to CEA, we also calculated the protein/carbohydrate, lipid/protein, and lipid/carbohydrate ratios for each sample.

We fit linear mixed models using the package *lme4* to assess the influence of treatment (fed vs. unfed) on each biomarker, including tray as a random effect in each model to account for the nonindependence of mussels collected from the same container (*N* = 10 samples per treatment; *n* = 3–4 samples per tray; Bates *et al.,* 2015). We used the package *lmerTest* to calculate the *t* statistics of the fixed effects, approximate the degrees of freedom using Satterthwaite’s method, and obtain *P* values (Kuznetsova *et al.,* 2017). Values of all constituent biomarkers, CEA, and the protein/carbohydrate ratio met assumptions of normality and homogeneity of variance. Lipid/protein and lipid/carbohydrate ratios were non-normally distributed, so we log-transformed them, which resulted in a normal distribution of values. We conducted these analyses in R version 4.1.3 (R Core Team, 2022).

### Experiment 2: assessing the effects of food availability and temperature on ETS activity

We cultured *L. cardium* glochidia from broodstock collected from the East Fork White River, Indiana, USA, on Largemouth Bass (*M. nigricans*) in March 2021 as described for Experiment 1. We reared the juvenile mussels for 5 months in a greenhouse with natural lighting at 24 to 26°C in 5.8-l aerated trays within a static aquaculture system. The trays, water source, and substrate were identical to those in Experiment 1. Because trays were static, we placed about 20 biological filter media in each tray (Bio-barrels or Bio-balls, Pentair, Cary, North Carolina, USA) and carried out a full, manual water change once daily. Additionally, every 8 h, an automated ball valve delivered a volume of water via gravity feed over 8 min, equivalent to approximately one-third of a water change, with excess water overflowing through a bulkhead fitting at the set water level into a floor drain. We fed the mussels the same ration as described previously, but the full ration was delivered to each tray once per day, after the water change was complete.

We conducted a 3$\times$3 full factorial experiment to evaluate the effects of food availability and temperature on mussel growth, survival, and physiological status. We conducted the experiment using the same static system described previously for culturing juveniles. We established three food ration levels: high, which was the standard ration fed to juvenile mussels during culture and in the fed treatment in Experiment 1 (see previous); low, which was 20% of the standard ration; and unfed, which received no food. We delivered food to each tray once per day as described previously. We established three temperature levels: 20, 25, and 30°C. This range encompasses temperatures frequently experienced by mussels in the eastern USA, including the minimum temperature for growth of many species (20°C), but it does not include temperatures at which heat stress begins to occur (>30°C; Carey *et al.,* 2013; Haag *et al.,* 2019; Fogelman *et al.,* 2023). Each unique treatment combination had two replicate trays such that *N* = 6 for each treatment main effect (food and temperature, *N* = 18 trays total). We placed all six trays for each temperature level in a water bath within a 56 × 120 × 18 cm plastic crate. We randomized the placement of trays within each temperature crate, and we randomized the placement of the three crates on the greenhouse shelving. We maintained temperatures in each water bath using a combination of heating mats, submersible aquarium heaters, and inline chillers. We recorded water temperature every 60 min in two trays in each temperature level using IBWetland temperature loggers (Alpha-Mach Inc., Sainte-Julie, Quebec, Canada). The mean temperature over the course of the experiment was within ±1°C of the target temperature in all treatment levels.

We placed 30 haphazardly selected juvenile mussels (mean initial individual blotted wet mass with shell = 0.018 g ± 0.007 SD; mean shell length = 5.0 mm ± 0.3 SD) in each tray on 7 September 2021, prior to bringing water baths to target temperatures. We brought temperatures to targets gradually over the next 24 h, and we considered September 8 as Day 1 of the experiment. We ran the experiment until 4 October 2021 (27 days). Upon termination of the experiment, we weighed all mussels in each tray (blotted wet mass), estimated mean individual mass by dividing the total mass by the number of surviving mussels, and froze all surviving mussels at −80°C. We expressed survival as proportional survival, based on the numbers of live individuals at the beginning and end of experiments. We expressed mussel growth as instantaneous growth, as described for Experiment 1.

We estimated ETS activity as described for Experiment 1, with the following exceptions. We thawed samples on ice and homogenized the whole body (shell + tissue) of each mussel with a mortar and pestle. Depending on the size of mussels, we prepared samples consisting of one to eight individuals from each experimental tray to achieve a minimum sample mass of 50 mg, and we estimated ETS activity for two to four samples per tray. We diluted the samples with reagent grade deionized water to a final concentration of 1 mg tissue·ml^−1^ homogenate, distributed the diluted homogenate from each sample into twenty 2-ml microcentrifuge tubes, and stored them at −80°C. We then thawed samples on ice and measured ETS activity at the corresponding treatment temperature for each sample (20, 25, or 30°C). We converted ETS from ‘mg O_2_·g^−1^·h^−1^’ to “mJ·mg^−1^·h^−1^’ as described for Experiment 1.

We used a two-way analysis of variance (ANOVA) to evaluate the effects of temperature, food, and the temperature × food interaction on growth and ETS activity; we evaluated separate models for each dependent variable (growth and ETS). ETS activity did not differ significantly among the 2–4 samples measured in each tray (nested ANOVA, tray [temperature × food], *F*_9,35_ = 1.64, *P* = 0.142), so we used mean ETS activity across samples in each tray (*N* = 18 observations) in the ANOVA model. We found no evidence for violation of assumptions of normality and homogeneity of variance for values of growth or ETS. Because a significant interaction precluded interpretation of experiment-wide main effects, we used the SLICE and CONTRAST procedures and Bonferroni adjustment of *P* value in SAS (version 9.4; SAS Institute, Cary, NC, USA) to test specific hypotheses about treatment effects. We conducted these analyses in SAS version 9.4 (SAS, 2013).

## Results

### Experiment 1: testing the ability of individual biomarkers and CEA to distinguish between fed and starved mussels

**Figure 1:**
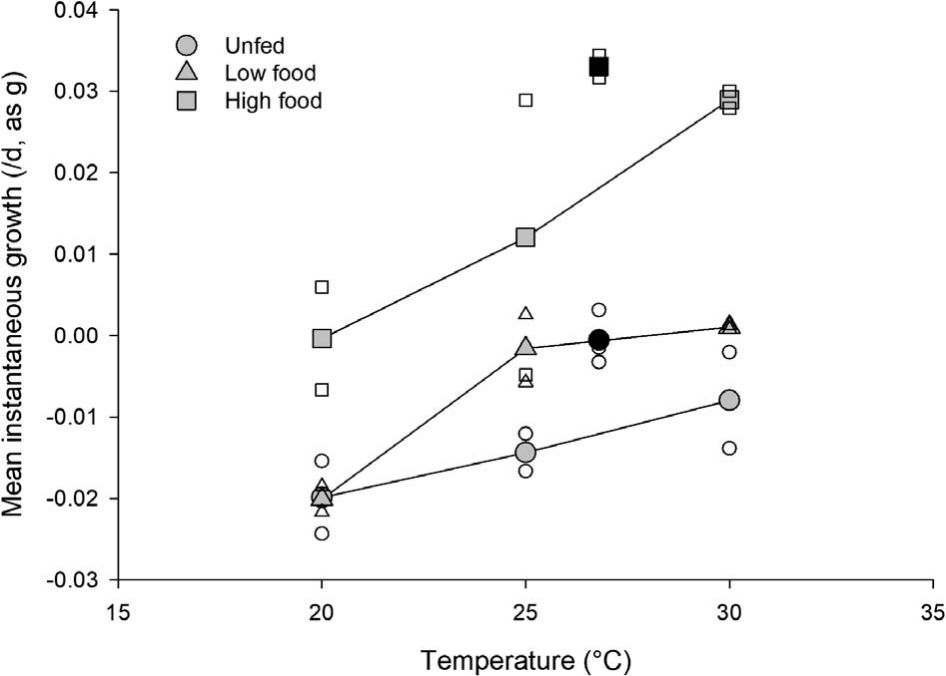
Mean instantaneous growth of juvenile mussels in relation to water temperature and food abundance in Experiments 1 and 2 over 23 days and 27 days, respectively. Filled black symbols indicate mean growth at 26.8°C for unfed and high food treatments in Experiment 1, filled gray symbols indicate mean growth in each treatment combination in Experiment 2, and open symbols represent raw data.

Survival was 100% in the fed treatment. Mortality was first observed in the unfed treatment on July 10 (day 20), and mortality continued until July 13 when the experiment was terminated. Cumulative instantaneous mortality in the unfed treatment ranged from −0.021 to −0.073·day^−1^ (85–90% survival over the course of the experiment). Mean instantaneous growth was 0.033·day^−1^ (as g) ± 0.001 SE in the fed treatment and −0.001·day^−1^ (as g) ± 0.002 SE in the unfed treatment (Fig. 1; increase in mass: fed, 0.059–0.066 g; unfed, −0.003 to 0.003 g).

**Figure 2:**
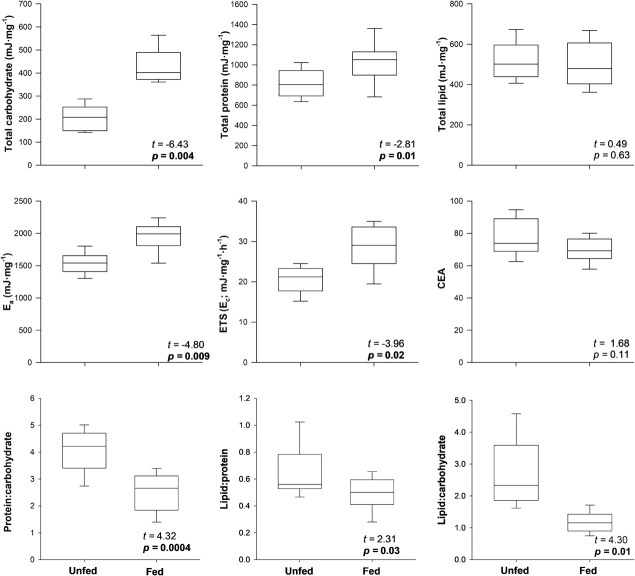
Boxplots showing CEA and constituent biomarkers of juvenile mussels in relation to food treatment in Experiment 1. *E*_a_ is available energy reserves (carbohydrate + protein + lipid), ETS is electron transport system enzyme activity (a proxy for energy consumption, *E*_c_), and CEA is *E*_a_/*E*_c_. Horizontal lines are medians, boxes are the 25^th^ and 75^th^ percentiles, and whiskers are 10th and 90th percentiles. Test statistics and *P* value are from linear mixed models, and significant differences (α = 0.05) are bolded.

Total carbohydrate, total protein, *E*_a_, and *E*_c_ (ETS) were greater in fed mussels than in unfed mussels (Fig. 2; Table S1). Lipid/protein, protein/carbohydrate, and lipid/carbohydrate ratios were greater in unfed mussels than in fed mussels. Total lipid and CEA did not differ between fed and unfed mussels.

### Experiment 2: assessing the effects of food availability and temperature on ETS activity

Survival was 100% in all food treatments at 20 and 25°C except for the 20°C low food treatment (mean survival = 98% ± 2 SE). At 30°C, mean survival was 70% (±1 SE) in the unfed treatment, 100% in the low food treatment, and 95% (±2) in the high food treatment. Temperature, food, and the temperature × food interaction all were significant factors in explaining variation in survival among treatments (ANOVA, *F*_2–4, 9_ = 106.58–178.40, *P* < 0.001 for all factors), but this result appeared to be driven by lower survival in the 30°C unfed treatment.

Mean instantaneous growth in the 30°C high food treatment [0.029·day^−1^ (as g) ± 0.001] was similar to growth in the fed treatment in Experiment 1, but growth varied widely among temperature and food treatments (Fig. 1). Temperature and food were significant factors in explaining variation in growth among treatments (ANOVA, temperature: *F*_2, 9_ = 7.27, *P* = 0.013; food: *F*_2, 9_ = 13.67, *P* = 0.002), but the temperature × food interaction was not significant (*F*_4, 9_ = 0.64, *P* = 0.648). Growth increased with increasing temperature (least-squares mean instantaneous growth per day, as g: 20°C = −0.0135; 25°C = −0.001; 30°C = 0.007) and with increasing food (unfed = −0.014; fed low = −0.007, fed high = 0.014).

**Figure 3:**
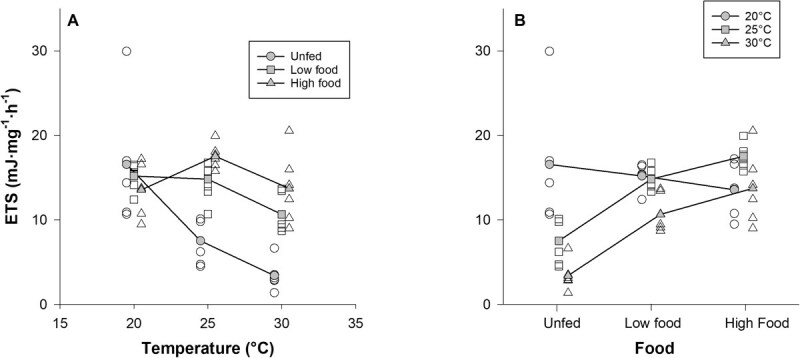
Interaction plots showing the effects of temperature × food on ETS enzyme activity of juvenile mussels in Experiment 2. (A) Effects of temperature at different food levels. (B) Effects of food at different temperature levels. Open symbols represent values from individual replicate trays, and filled symbols represent mean values for each treatment combination.

Both temperature and food were significant factors in explaining variation in ETS activity (ANOVA, temperature: *F_2,9_* = 8.60, *P* = 0.008; food: *F_2,9_* = 10.11, *P* = 0.005), and ETS activity decreased overall with increasing temperature and increased with increasing food abundance (Fig. 3; Table S2). However, the temperature × food interaction also was significant (*F_4,9_* = 4.55, *P* = 0.028). Contrasts showed that ETS activity was affected by temperature only in the unfed treatment (*F*_2,5_ = 13.82, adjusted *P* = 0.005) and not in either fed treatment (*F*_2,5_ = 10.15–12.73, adjusted *P* = 0.515–0.698). Contrasts showed that ETS activity was affected by food at 25 and 30°C (*F*_2,5_ = 9.11–9.58, adjusted *P* = 0.018–0.021) but not at 20°C (*F*_2,5_ = 0.51, adjusted *P* = 1.000).

## Discussion

Both of our experiments were of sufficient duration to induce stress in response to low food availability. Although interpretation of mussel growth as a stress biomarker in the wild can be confounded by environmental factors, reduced growth represents a clear stress response in controlled laboratory experiments or field studies with limited environmental variation (Haberle *et al.,* 2025; Pankratz *et al.,* 2025). We observed negative or only very low growth in no food and low food treatments. Growth in high food treatments was similar to that observed in warm, productive streams in Kentucky, USA (Haag *et al.,* 2019). Similar to previous studies, temperature had a strong effect on growth, and little growth occurred at 20°C in any food treatment (Carey *et al.,* 2013; Haag *et al.,* 2019). However, we found no evidence that food availability affected the relationship between growth and temperature over the duration of our experiments. The onset of mortality near the end of Experiment 1 suggested that stress was severe enough to result in death at ≥20 days in the unfed treatment.

A reduction in energy reserves is a common response to stress in aquatic ectotherms (see Sokolova, 2021 and sources therein). In Experiment 1, total energy reserves (*E*_a_) were reduced in unfed mussels, but components of *E*_a_ responded differently. Starvation resulted in a ~50% depletion of short-term energy reserves (total carbohydrate) over 23 days compared to fed mussels. A similar magnitude of glycogen depletion was observed in adult mussels after 14–30 days of starvation in the laboratory (Patterson *et al.,* 1999) and after 4 months of fouling by *Dreissena polymorpha* (which apparently competed for food with native mussels, Haag *et al.,* 1993). Starvation also resulted in a depletion of total protein, but to a lesser extent than carbohydrate (about 30% lower than fed mussels), which was supported by the higher protein/carbohydrate ratio in unfed mussels. Starvation resulted in no detectable depletion of lipid reserves, along with higher lipid/protein and lipid/carbohydrate ratios in unfed mussels. Based on our results and other studies on marine and freshwater bivalves, lipids appear to be long-term energy reserves that are conserved during short-term starvation or food limitation (Haag *et al.,* 1993; Pleissner *et al.,* 2012; Supono *et al.,* 2023). However, the onset of mortality in unfed mussels near the end of Experiment 1 suggests that lipid reserves alone are insufficient to meet the energetic needs of juvenile mussels, and a decline in overall *E*_a_ is ultimately lethal.

The range of ETS values we observed in both experiments was similar to that observed in a wild population of *D*. *polymorpha* in Lake Huron from April to November (Fanslow *et al.,* 2001). ETS activity (*E*_c_) was lower in unfed mussels than in fed mussels in Experiment 1, which may represent lower energy consumption associated with reduced filtering activity due to food limitation (see subsequent). In Experiment 2, ETS activity was responsive to food and temperature but only under some conditions. Like Experiment 1, unfed mussels had lower ETS activity than fed mussels, but only at 25 and 30°C, and there was no effect of food on ETS at 20°C. For unfed mussels, there was a reduction in ETS activity with increasing temperature, but there was no effect of temperature on ETS in fed mussels. These responses show that ETS responds in complex ways to stressors such as food limitation as well as environmental variables such as temperature.

It is expected that ETS activity will increase with increasing temperature, at least up to temperatures that result in heat stress (~30°C, Fogelman *et al.,* 2023). Mussel respiration rate increases linearly or exponentially from 20 to 30°C (Lomte and Nagabhushanam, 1971; Ganser *et al.,* 2015; Jeffrey *et al.,* 2018; Fluharty *et al.,* 2023), and total energy reserves declined sharply between 18 and 30°C, indicating greater energetic needs at higher temperatures (Said and Nassar, 2022). Accordingly, ETS increased with temperature in *D*. *polymorpha* (Fanslow *et al.,* 2001), but over a greater range of temperatures (5–25°C) than in our study. In Experiment 2, higher mortality in the unfed treatment at 30°C than at 20 and 25°C suggests that greater metabolic demands at higher temperatures depleted energy reserves more quickly (see Haney *et al.,* 2020). However, we found no effect of temperature on ETS in fed mussels and a negative effect in starved mussels. Furthermore, food had no effect on ETS at 20°C, unlike its strong effects at 25–30°C in both experiments. ETS activity increased with temperature in the mussel *Sinanodonta woodiana*, but not in *Anodonta anatina*, for which the magnitude of ETS across temperatures was similar to that seen in our study (Bielen *et al.,* 2016). In addition to temperature, ETS responded differently to zinc exposure in *S*. *woodiana* and *A*. *anatina*, and other studies have found complex ETS responses in bivalves related to temperature, species identity, and other confounding factors (Gagné *et al.,* 2007; Crespo *et al.,* 2021).

The response of ETS has a large influence on the usefulness of CEA for evaluating stress. Energy consumption (*E*_c_) can increase with stress due to higher metabolic demands, in which case reduced energy reserves (*E*_a_) will result in large declines in CEA (e.g. De Coen and Janssen, 2003). In bivalves, reduced energy consumption associated with valve closure and a reduction of filter feeding is a common response to a wide variety of stressors (Anestis *et al.,* 2007; Tran *et al.,* 2007; Tang and Riisgård, 2016; Haney *et al.,* 2020; Fogelman *et al.,* 2022). In oysters and *D*. *polymorpha*, CEA decreased in response to pollutant exposure because *E*_a_ declined dramatically, but *E*_c_ declined to a lesser extent or remained unchanged (Smolders *et al.,* 2004; Bartlett *et al.,* 2020). In our study, CEA was unresponsive to starvation because *E*_c_ declined by approximately the same magnitude as *E*_a_.

Measurements of ETS activity are interpreted as a proxy for energy consumption as dictated by the organism’s maximum metabolic or respiration rate (Verslycke *et al.,* 2004). ETS is highly correlated with respiration rate in *D*. *polymorpha*, but seasonal or spatial variation in food quantity or quality or other factors can result in a mismatch between those measures (Madon *et al.,* 1998; Fanslow *et al.,* 2001). Differences between ETS and respiration rate are attributed to the more rapid response of respiration to changing environmental conditions than ETS, for which a response requires changes to the existing mitochondrial enzymatic machinery; therefore, respiration rate represents actual, instantaneous oxygen consumption, while ETS represents potential maximum oxygen consumption integrated over the previous several weeks (Packard, 1971; Båmstedt, 1980; Fanslow *et al.,* 2001). In *D*. *polymorpha* exposed to pollutants, changes in ETS lagged at least 1 week behind changes in respiration rate, which may partially explain the lower-magnitude ETS response to stress compared with energy reserves in that study (Smolders *et al.,* 2004).

In our study, traditional biomarkers such as carbohydrates and protein, as well as ratios of *E*_a_ components, were responsive and informative indicators of stress induced by starvation. ETS activity was informative but only at ≥25°C, and its response to temperature varied according to food abundance. As a biomarker, ETS activity remains potentially valuable in some situations, but confounding factors such as food, temperature, and species identity, as well as the lag time in response of ETS relative to respiration rate, need to be accounted for. Despite its value as a robust, holistic stress biomarker in other organisms, CEA may have limited usefulness for bivalves because of their tendency to reduce feeding and energy consumption under stress, which results in a simultaneous decline in *E*_a_ and *E*_c_. Studies of CEA responses to a wider array of stressors are needed to more fully evaluate its usefulness as a stress biomarker in mussels.

## Supplementary Material

Web_Material_coaf086

## Data Availability

The data that support the findings of this study are available from the corresponding author, K.J.F., upon reasonable request.
