# Supplementary material for: Utility of the cellular energy allocation model for assessing food limitation stress in freshwater mussels
Source: Conserv Physiol. 2025 Dec 18;13(1):coaf086. doi: 10.1093/conphys/coaf086 (PMC12712612; doi:10.1093/conphys/coaf086)
Supplement: Web_Material_coaf086 [file web_material_coaf086.zip › Juvenile_Mussel_CEA_supp_tables_for_submission (1).pdf]

Supplementary Tables

S1. Mean values (± SE) for biomarkers related to cellular energy allocation (CEA) in fed and unfed juvenile *Lampsilis cardium*. E_a is energy available (sum of carbohydrates, protein, and lipids) and E_c is energy consumption (measured via electron transport system enzyme activity). All values are $mJ\cdot mg^{-1}$ except E_c ($mJ\cdot mg^{-1}\cdot h^{-1}$), CEA, and ratios (no units). The columns t and p are results of linear mixed models between fed and unfed mussels for each biomarker. Comparisons with $p < 0.05$ are bolded.

	**Mean (± SE)**				
**Biomarker**	**Unfed**	**Fed**	***df***	***t***	***p***
Total carbohydrates	209.1 (16.6)	429.9 (21.6)	3.65	-6.43	**0.004**
Total protein	818.2 (43.6)	1032.9 (62.8)	18	-2.81	**0.01**
Total lipids	520.3 (29.7)	497.9 (34.2)	18	0.49	0.63
E_a	1547.6 (51.4)	1960.7(67.6)	3.96	-4.80	**0.009**
E_c	20.4 (1.0)	28.6 (1.7)	4.18	-3.96	**0.02**
CEA	76.9 (3.6)	69.6 (2.4)	18	1.68	0.11
Protein:carbohydrate ratio	4.1 (0.25)	2.5 (0.23)	18	4.32	**0.0004**
Lipid:protein ratio	0.66 (0.06)	0.50 (0.04)	18	2.31	**0.03**
Lipid:carbohydrate ratio	2.7 (0.35)	1.2 (0.10)	3.73	4.30	**0.01**

S2. Juvenile *Lampsilis cardium* mean electron transport system (ETS) activity ($mJ\cdot mg^{-1}\cdot h^{-1}$) ± standard error (SE) under feeding treatments, of unfed, low, or high food; temperatures of 20, 25, and 30°C and interactions between food and temperature.

**Effect**	**Variables**	**Mean (± SE)**
Food	Unfed	8.73 (1.70)
	Low Food	13.31 (0.64)
	High Food	14.95 (0.82)
Temperature	20 °C	15.02 (1.18)
	25 °C	13.30 (1.11)
	30 °C	9.34 (1.17)
Food × Temperature	Unfed × 20 °C	16.58 (3.54)
	Low Food × 20 °C	15.2 (0.74)
	High Food × 20 °C	13.57 (1.25)
	Unfed × 25 °C	7.52 (1.09)
	Low Food × 25 °C	14.83 (0.52)
	High Food × 25 °C	17.55 (0.59)
	Unfed × 30 °C	3.40 (0.71)
	Low Food × 30 °C	10.66 (0.79)
	High Food × 30 °C	13.73 (1.71)
